# Antagonizing urotensin receptor is a novel therapeutic strategy for glucocorticoid‐induced skeletal muscle atrophy

**DOI:** 10.1002/ctm2.879

**Published:** 2022-05-23

**Authors:** Lin Yin, Na Li, Weihua Jia, Nuoqi Wang, Meidai Liang, Jiamin Shang, Guifen Qiang, Guanhua Du, Xiuying Yang

**Affiliations:** ^1^ State Key Laboratory of Bioactive Substance and Function of Natural Medicines and Beijing Key Laboratory of Drug Target and Screening Research Institute of Materia Medica of Peking Union Medical College Beijing P. R. China

Dear editor,

A severe complication of glucocorticoids is skeletal muscle atrophy, but there are still no effective drug and target. We reported for the first time that a G protein‐coupled receptor—urotensin receptor (UT)—is a novel potential drug target against glucocorticoids‐induced skeletal muscle atrophy.

In clinical practice, glucocorticoids such as dexamethasone (Dex) are commonly used to treat many conditions caused by immunologic disorders, inflammation, etc.^1^ However, glucocorticoids can also bring side and even life‐threatening effects including skeletal muscle atrophy,^2^ which leads to great inconvenience to people's everyday lives and increases the mortality and disability.^3^ Therefore, drugs against glucocorticoids‐induced muscle atrophy have clinical significance.^4^ Unfortunately, no drugs are currently used in clinical practice so far.

UT is the receptor for the endogenous polypeptide urotensin‐II (U‐II). Both of them are widely distributed in various tissues including skeletal muscles. U‐II is known as the strongest endogenous vasoconstrictive polypeptide.^5^ UT is involved in cardiovascular diseases, metabolism, and chronic inflammation.^6^ However, there are still no drugs targeting UT on the market due to the limited therapeutic effect on current indications. Our previous studies have found that UII regulates energy metabolism in skeletal muscle.^7^ Recently, Pan et al.^8^ pointed that U‐II induces skeletal muscle atrophy in mice with chronic renal failure, but the mechanism is unknown.

To decipher whether urotensinergic system is associated with the muscle atrophy caused by glucocorticoids, male C57BL/6 mice were subjected to Dex (25 mg/kg/day) (Figure 1A), where the muscle strength (Figure 1B) and mass (Figure 1C) were lower, and muscle atrophic markers increased (Figure 1D), while myosin heavy chain (*Myh1*) that stands for the myocyte skeleton dramatically decreased in different types of muscles (soleus, extensor digitorum longus and gastrocnemius, Figure 1D). To our surprise, the levels of U‐II in blood (Figure 1E) and the *Uts2* expression in muscles (Figure 1F) were all elevated due to Dex.

**FIGURE 1:**
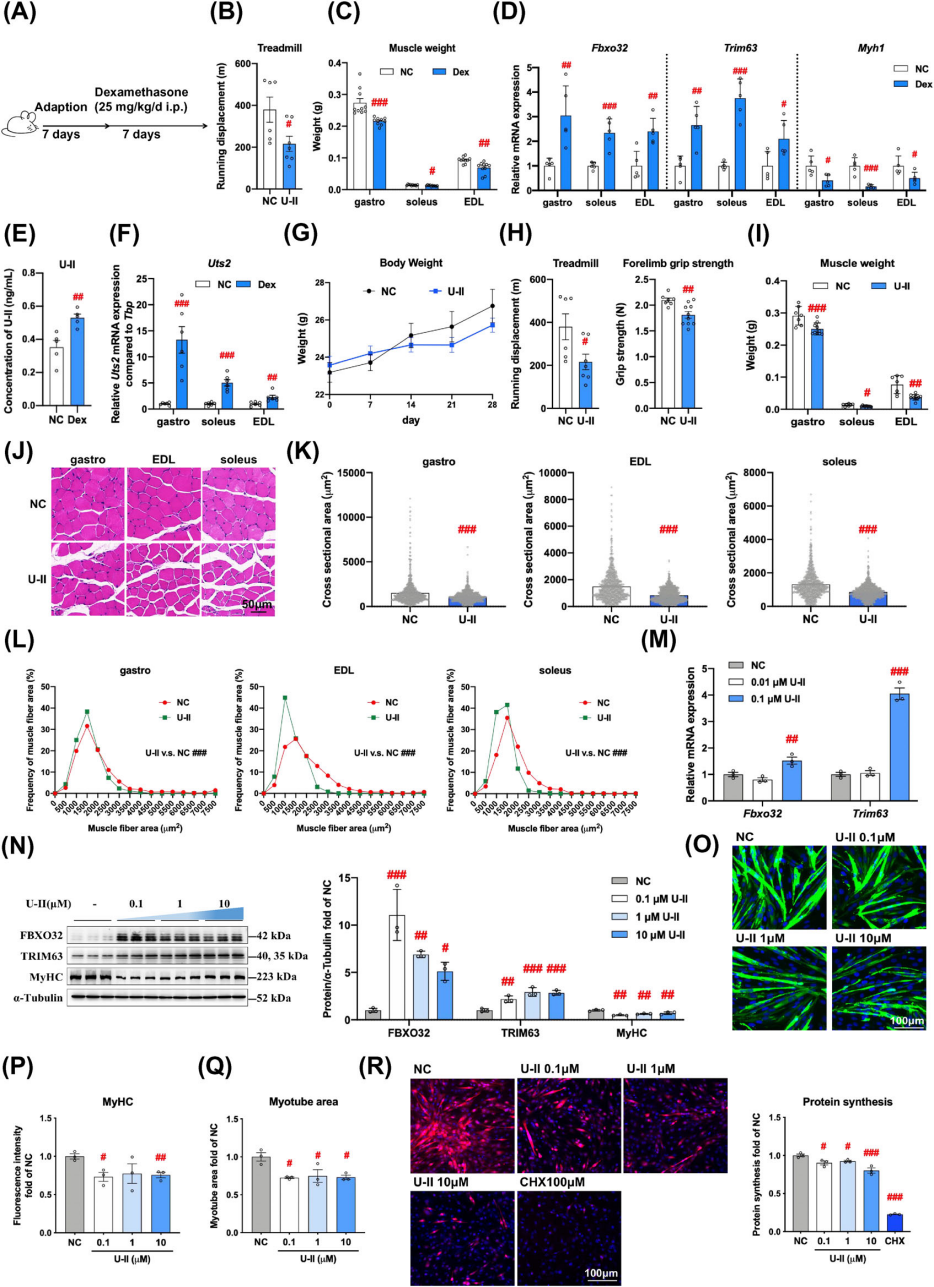
Dex induces muscle atrophy with elevated U‐II production in mice, and U‐II can lead to muscle atrophy in mice and myotubes. (A–F) Male C57/BL/6 mice were subjected to Dex to establish model of muscle atrophy. (A) Schematic diagram. (B) Treadmill test after Dex modeling. (C) Muscle weights. (D) messenger RNA (mRNA) expression levels. (E) Concentration of U‐II in plasma. (F) mRNA expressions of *Uts2*. (G–L) Male C57/BL/6 mice were treated with U‐II (50 μg/kg/day i.p.) for 4 weeks. (G) Changes of body weight. (H) Treadmill tests and forelimb grip strengths. (I) Muscle weights. (J) Hematoxylin   Eosin (HE) stains (200x magnification). (K) Cross‐sectional areas. (L) Frequency distributions. (M–R) Mouse C2C12 myotubes were treated with U‐II for 48 h. (M) Expressions of *Fbxo32* and *Trim63* at mRNA levels. (N) Immunoblot analyses of FBXO32, TRIM63 and MyHC. (O) Immunofluorescence stain of MyHC protein in C2C12 myotubes after 48‐h U‐II treatment (100x magnification). (P) Fluorescence intensity of MyHC normalized with nucleus. (Q) Areas of the myotubes. (R) Protein synthesis in C2C12 myotubes after 48‐h U‐II treatments. *n* = 5‐15 (A–L), or *n* = 3 (M–R); means ± standard error of mean (SEM); *p* values less than: .05 (#), .01 (##), .001 (###) compared with normal control (NC)

We then accessed U‐II on skeletal muscle at the cellular and animal levels. Male C57BL/6 mice were given U‐II (50 μg/kg/day i.p. for 4 weeks, Figure S1A). We found U‐II caused a drastic attenuation of muscle strengths (Figure 1H), weights (Figure 1I) and the cross‐sectional areas (Figure 1K), and the smaller areas increased in diverse types of muscles (Figure 1L). Further, the results in vitro showed U‐II (0.1, 1, 10 μM, 48 h) dramatically increased muscle atrophic markers in myotubes at both messenger RNA (mRNA) and protein levels (Figure 1M, N). Meanwhile, U‐II decreased myosin heavy chain (MyHC) (Figure 1N), and immunofluorescence stain yielded similar results (Figure 1O,P). In addition, the myotube areas shrank (Figure 1Q) resembling with the decreased cross‐sectional areas and muscle masses seen in the U‐II treated mice. Besides, protein synthesis was inhibited by U‐II (Figure 1R). These findings show U‐II is an inducing factor and sufficient for muscle atrophy both in vivo and in vitro.

The above results led to an important question, which was whether the receptor of U‐II—UT is a drug target for Dex‐induced muscle atrophy. We then generated the *Uts2r* global knockout mice (Figure 2A), which were subjected to Dex (Figure 2C). We found *Uts2r* knockout effectively protected against muscle atrophy triggered by Dex as evidenced by improvement in muscle strengths (Figure 2D) and masses (Figure 2E). Furthermore, the cross‐sectional areas of muscles were all improved (Figure 2G), and the frequency distributions were biased toward larger area (Figure 2H).

**FIGURE 2:**
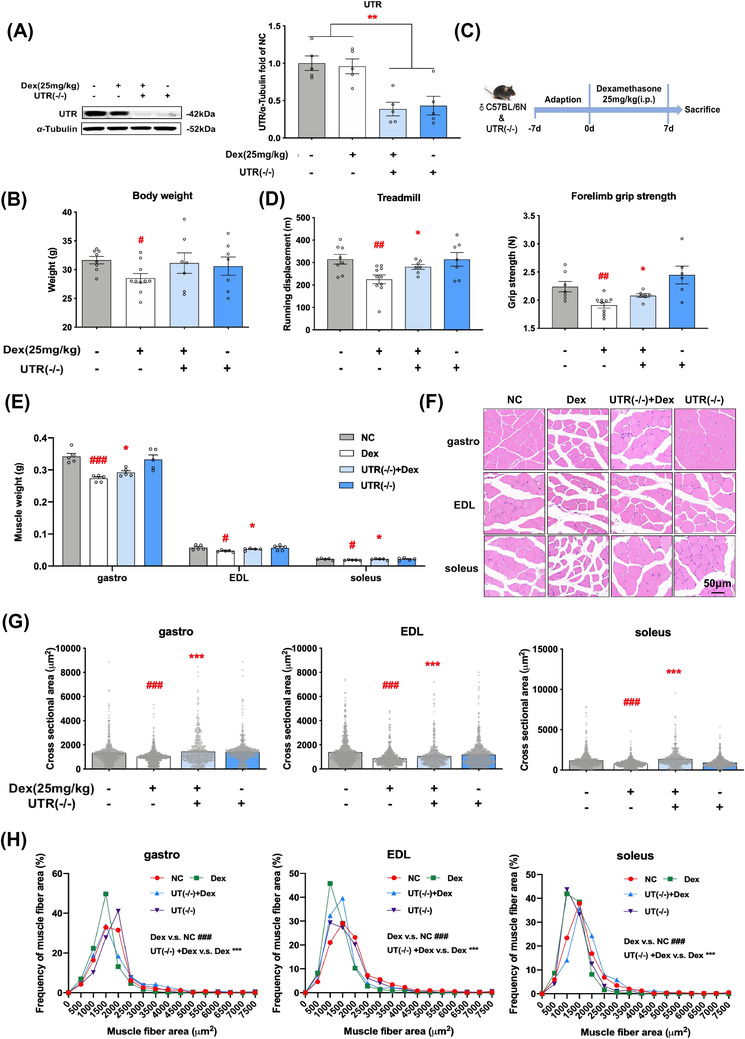
Knockout of *Uts2r* alleviates Dex‐induced skeletal muscle atrophy in vivo. Normal male C57/BL/6 mice and *Uts2r* knockout mice were subjected to Dex. (A) Identification of knockout of *Uts2r*. (B) Change of body weights. (C) Schematic diagram of administration. (D) Treadmill tests and forelimb grip strengths. (E) Muscle weights of gastro, extensor digitorum longus (EDL) and soleus. (F) Hematoxylin   Eosin (HE) stains (200x magnification). (G) Cross‐sectional areas. (H) Frequency distributions of muscle fibers. *n* = 5‐10. Means ± standard error of mean (SEM). *p* values less than: .05 (#), .01 (##), .001 (###) compared with normal control (NC); .05 (*), .01 (**), .001 (***) compared with Dex group

To verify whether UT antagonism has the effect of alleviating muscle atrophy induced by Dex, we explored the effect of palosuran (a non‐peptide antagonist of UT receptor) in mice. Male C57BL/6 mice were injected with Dex then followed with 2‐week palosuran treatments. Results showed muscle strengths (Figure 3A) and tissue indexes of tibialis anterior and gastrocnemius (Figure 3B) were significantly enhanced in palosuran‐treated mice. In addition, the cross‐sectional areas of muscles all shrank in Dex group, and palosuran reversed this trend (Figure 3D) with the frequency distributions tending to be larger (Figure 3E). Consistently, in vitro study showed that palosuran (10, 100 and 1000 nM) ameliorated Dex‐induced myotube atrophy as determined by the decrease of muscle atrophic markers (Figure 3G) and improvement of myotube areas (Figure 3H‐J) as well as protein synthesis (Figure 3K) in Dex‐treated myotubes.

**FIGURE 3:**
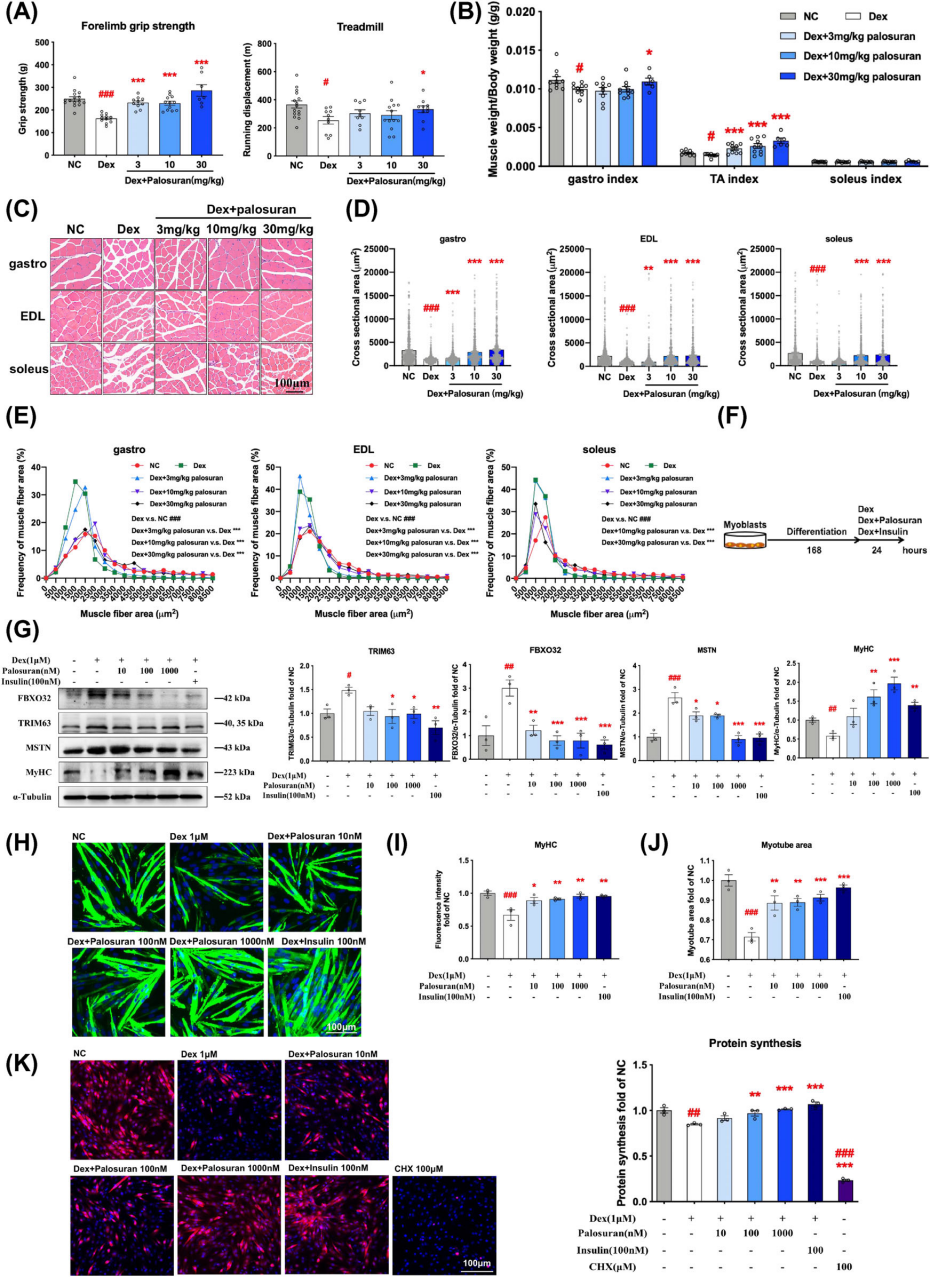
Antagonizing urotensin receptor (UT) alleviates Dex‐induced skeletal muscle atrophy in myotubes and mice. (A–E) Male C57/BL/6 mice were subjected to 7‐d Dex followed by 14‐d palosuran treatments. (A) Forelimb grip strengths and treadmill tests after 2‐week administration of palosuran. (B) Muscle indexes (muscle weight/body weight). (C) Muscle Hematoxylin   Eosin (HE) stains (200x magnification). (D) Cross‐sectional areas of muscle fibers. (E) Frequency distributions of muscle fibers. (F–K) 24‐h palosuran on C2C12 myotubes with 1 μM dexamethasone. (F) Schematic diagram of the experiment processing. (G) Immunoblot analyses of FBXO32, TRIM63, MSTN and MyHC. (H) Immunofluorescence stains of MyHC in C2C12 myotubes (100x). (I) The relative fluorescence intensity of MyHC normalized with the fluorescence intensity of nucleus. (J) The areas of the myotubes. (K) Protein synthesis levels in C2C12 myotubes. Means ± standard error of mean (SEM). *n* = 6–15 (A–E); or *n* = 3 (F–K). *p* values less than: .05 (#), .01 (##), .001 (###) compared with normal control (NC); .05 (*), .01 (**), .001 (***) compared with Dex group

Mechanism investigations showed that PI3K/protein kinase B (AKT)/the mammalian target of rapamycin (mTOR) pathway was down‐regulated by Dex (Figure S4A, B) as well as U‐II (Figure 4A,B) treatment. Palosuran, instead, retrieved the deleterious expression trend triggered by Dex and increased expressions of myofibrillar proteins in vitro (Figure S4A, B). Consistently, results in vivo revealed that palosuran remarkably activated PI3K/AKT/mTOR pathway, contributing to elevated expressions of myofibrillar proteins (Figure 4C,D). On the other hand, ubiquitin‐proteasome pathway was activated by Dex but ameliorated by palosuran (Figure 4E,F). Besides, the activity of succinate dehydrogenase and cytochrome oxidase, two mitochondrial marker enzymes,^9^, ^10^ were notably suppressed by Dex but restored by palosuran (Figure S4C), indicating that the therapeutic effect of palosuran is related to enhancing protein synthesis, attenuating ubiquitin‐proteasome pathway, and regulation of mitochondrial activity.

**FIGURE 4:**
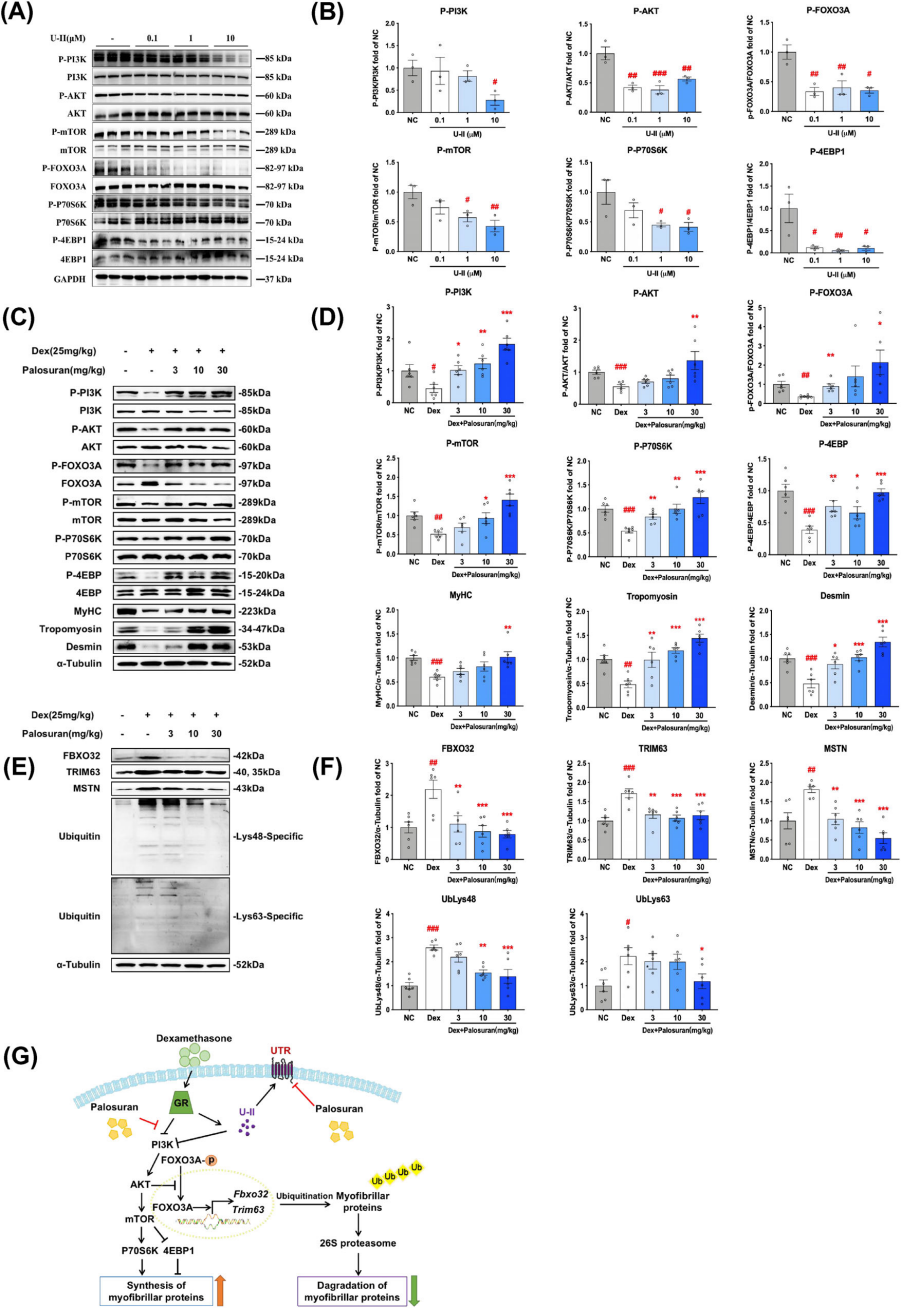
U‐II/palosuran mediate PI3K/protein kinase B (AKT)/the mammalian target of rapamycin (mTOR) and ubiquitin‐proteasome pathway regulating skeletal muscle. (A and B) After 48‐h U‐II treatments on C2C12 myotubes, the Western Blot results of myotubes. C‐F, C57/BL/6 male mice were administered 7‐d Dex followed by 14‐d palosuran treatments. (C and D) Proteins in PI3K signaling pathway in gastrocnemius. (E and F) Protein degradation‐related proteins in gastrocnemius. (G) Proposed signaling mechanisms. Means ± standard error of mean (SEM). *n* = 3 (A and B) or *n* = 6 (C–F). *p* values less than: .05 (#), .01 (##), .001 (###) compared with normal control (NC); .05 (*), .01 (**), .001 (***) compared with Dex group

In conclusion, our study reported for the first time that antagonizing UT receptor would be a novel therapeutic strategy for Dex‐induced skeletal muscle atrophy. We found that Dex induced elevated expression of U‐II, which could directly induce skeletal muscle atrophy. Knockout of *Uts2r* as well as UT receptor antagonism improved muscle atrophy triggered by Dex, which may be via increasing PI3K/AKT/mTOR and inhibiting ubiquitin‐proteasome pathways. These findings provide an experimental basis for UT as a potential drug target for glucocorticoid‐induced skeletal muscle atrophy.

## CONFLICT OF INTEREST

The authors declare that they have no competing interest.

## Supporting information

Supporting Information
