# Supplementary material for: Antagonizing urotensin receptor is a novel therapeutic strategy for glucocorticoid‐induced skeletal muscle atrophy
Source: Clin Transl Med. 2022 May 23;12(5):e879. doi: 10.1002/ctm2.879 (PMC9126497; doi:10.1002/ctm2.879)
Supplement: Supplementary file 1 — Supporting Information [file CTM2-12-e879-s001.pdf]

Supporting information

Additional supporting information may be found in the online version of the article at the publisher's website.

FIGURE S1

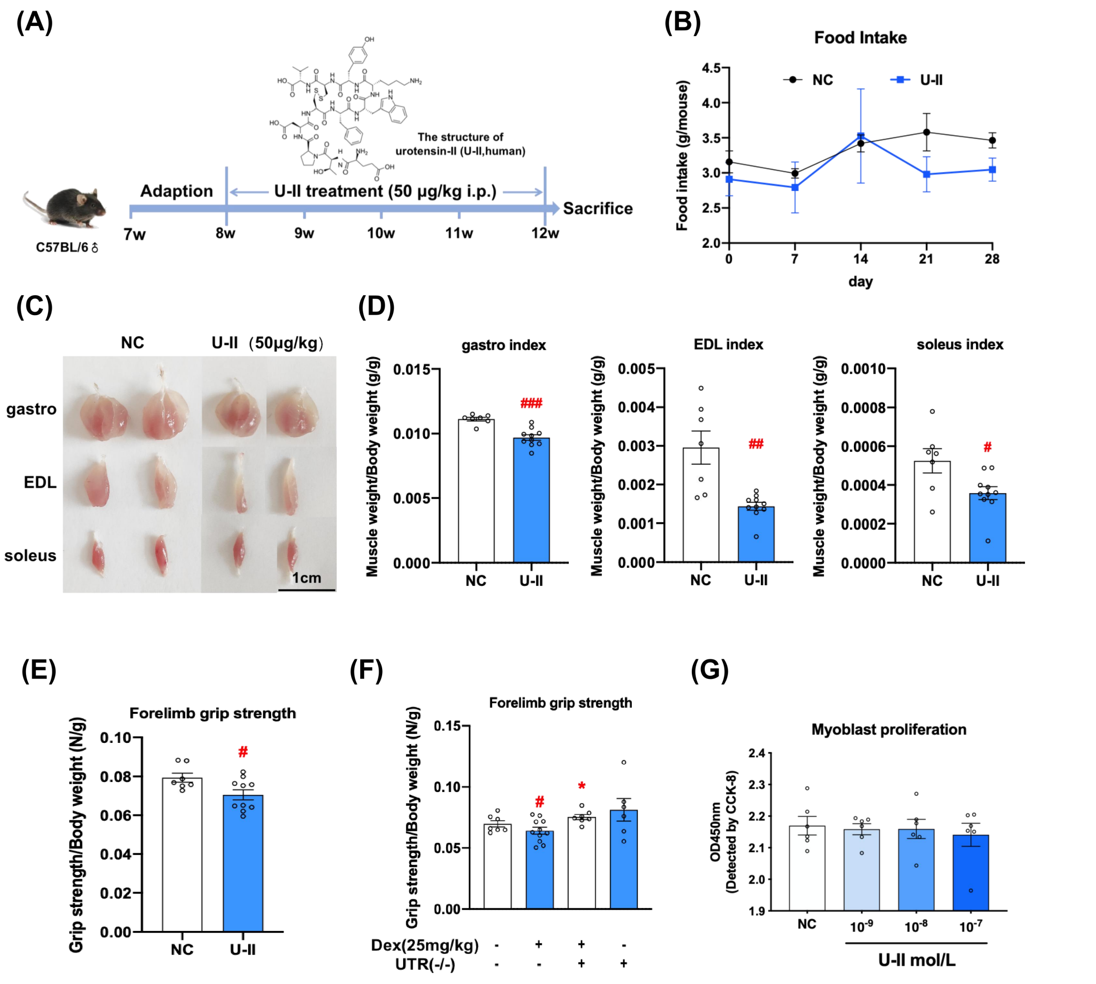

FIGURE S1 U-II regulates muscle weights and strength in mice, and UT knockout mice resists strength decrease induced by Dex. (A) A schematic diagram of the U-II administration. (B) Food intake of mice treated with U-II. (C) Represent morphology of muscles of mice. (D) Muscle weight indexes. (E) Forelimb grip strengths of U-II-treated mice. (F) Forelimb grip strengths of UT knockout mice. (G) Effect of U-II on myoblast proliferation. Means ± SEM, n=7-10. p values less than: .05 (#), .01 (##), .001 (###) compared with NC.

FIGURE S2

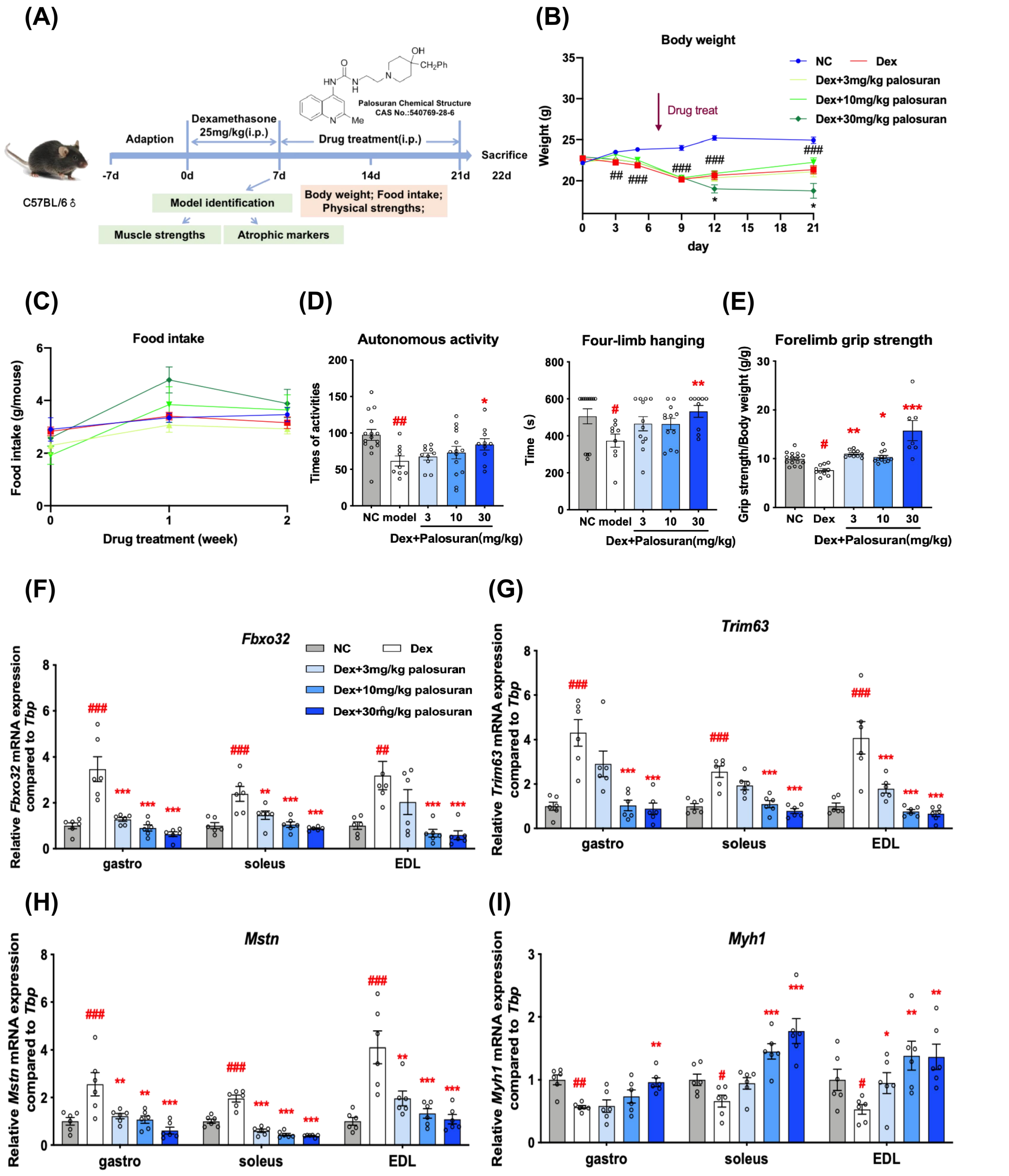

FIGURE S2 Palosuran alleviates dexamethasone-induced skeletal muscle atrophy *in vivo*. (A) A schematic diagram of the Dex administration followed by two-week palosuran treatment. (B) Time course of body weight changes. (C) Food intake. (D) Autonomous activity and four-limb hanging time after 2-week administrations of palosuran. (E) Forelimb grip strengths. (F-I) mRNA levels. (F) *Fbxo32*. (G) *Trim63*. (H) myostatin (*Mstn*). (I) *Myh1*. Means ± SEM. n=6-15. p values less than: .05 (#), .01 (##), .001 (###) compared with NC; .05 (*), .01 (**), .001

(***) compared with Dex group.

FIGURE S3

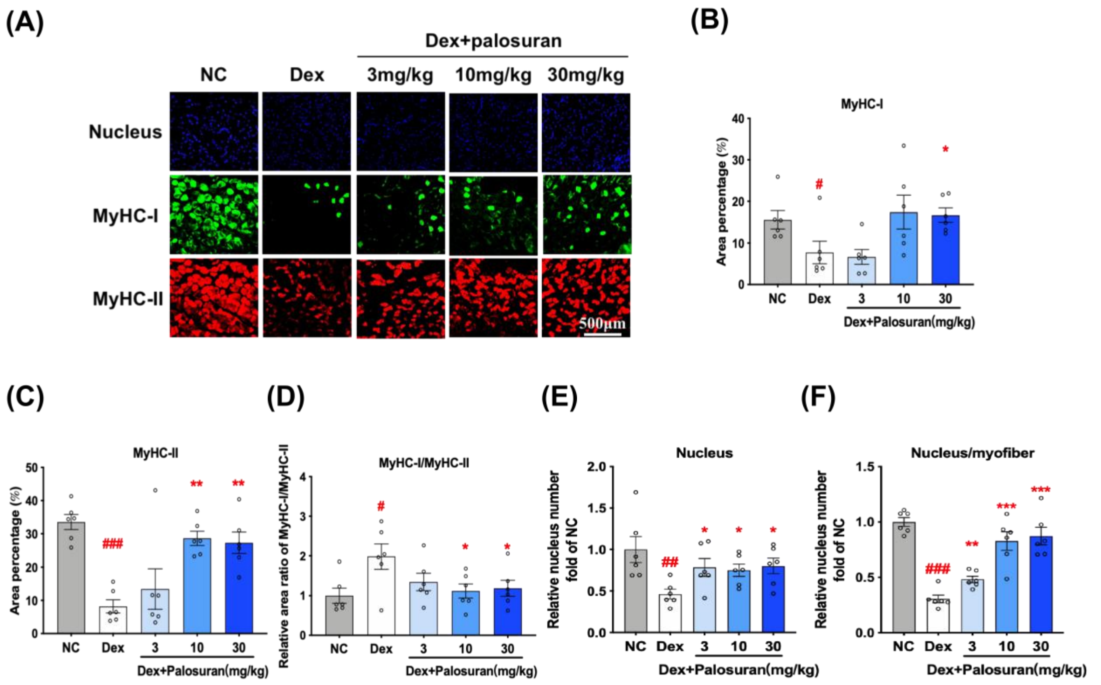

FIGURE S3 Palosuran ameliorates transformation of fiber types from fast- (MyHC-II) to slow-twitch (MyHC-I). (A) Representative images of immunofluorescence stain of nucleus, MyHC-I and MyHC-II (50 x magnification). (B) Area percentages (MyHC-I). (C) Area percentages (MyHC-II). (D) Relative area ratio of MyHC-I to MyHC-II. (E) Relative number of nuclei (normalized to myofibrillar area). (F) Relative number of nuclei (normalized to myofibrillar number). Means ± SEM. n=6. p values less than: .05 (#), .01 (##), .001 (###) compared with NC; .05 (*), .01 (**), .001 (***) compared with Dex group.

FIGURE S4

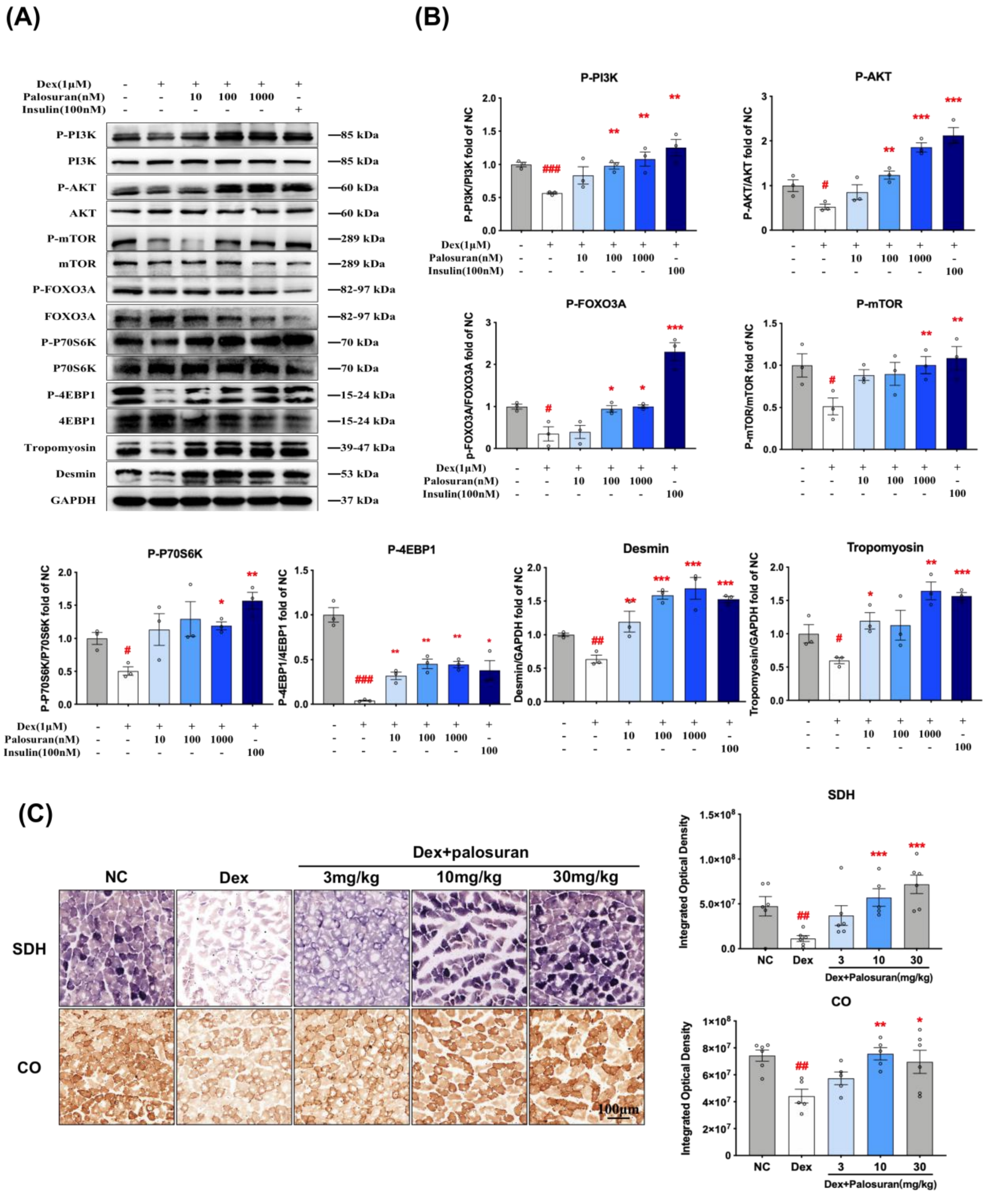

FIGURE S4 Palosuran mediates PI3K/AKT/mTOR pathway and regulates mitochondrial activity. (A-B) Expression analysis by Western Blot of C2C12 myotubes treated with 24-h palsoruan and 1 μM dexamethasone. (C) C57/BL/6 male mice were treated with Dex for 7 days and then were administered palosuran for 14 days, then the frozen sections of gastrocnemius were subject to SDH and CO stains (200 x magnification). Means ± SEM. n=3

(A-B) or n=6 (C). p values less than: .05 (#), .01 (##), .001 (###) compared with NC; .05 (*), .01 (**), .001 (***) compared with Dex group.

FIGURE S5

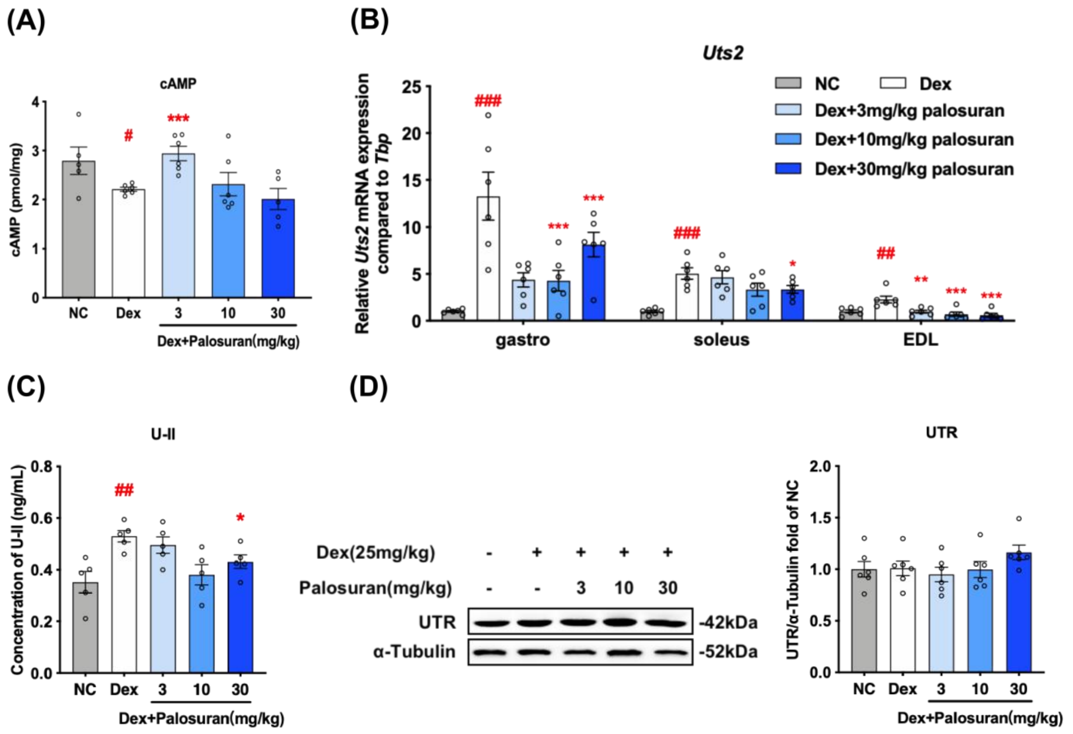

FIGURE S5 Palosuran treatment increases cAMP and decreases Dex-induced U-II levels in mice. C57/BL/6 male mice were treated with Dex for 7 days and then were administered palosuran for 14 days. (A) Concentration of cAMP in quadriceps. (B) Expressions of *Uts2 mRNA* in muscles. (C) Concentration of U-II in plasma. (D) Western Blot for UT. Means ± SEM. n=5-6. p values less than: .05 (#), .01 (##), .001 (###) compared with NC; .05 (*), .01 (**), .001 (***) compared with Dex group.

TABLE S1 Primers

Gene name	Species	Sequence (5' to 3')
Tbp	mouse	Forward:5'-ACCCTTCACCAATGACTCCTATG-3'
		Reverse:5'-ATGATGACTGCAGCAAATCGC-3'
Fbxo32	mouse	Forward:5'-CAGCTTCGTGAGCGACCTC-3'
		Reverse:5'-GGCAGTCGAGAAGTCCAGTC-3'
Trim63	mouse	Forward:5'-ACGAGAAGAAGAGCGAGCTG-3'
		Reverse:5'-CTTGGCACTTGAGAGAGGAAGG-3'
Myh1	mouse	Forward:5'-CATCCCTAAAGGCAGGCTCT-3'
		Reverse:5'-ATGCAGGTGGGTCATCATGG-3'
Mstn	mouse	Forward:5'-AGTGGATCTAAATGAGGGCAGT-3'
		Reverse:5'-GTTTCCAGGCGCAGCTTAC-3'
Foxo3a	mouse	Forward:5'-GAGCTGGAGCTCGAACCTT-3'
		Reverse:5'-GGGTGATCAGGTCGGCATAG-3'
Uts2	mouse	Forward:5'-GAGAAGCAGGTCCCAGCACT-3'
		Reverse:5'-AGCCCCGTGTTGCTTATGTT-3'

TABLE S2 Antibodies

Antibody	Supplier	Item NO.
PI3K	Cell Signaling Technology	4257
Phospho-PI3K	Immunoway	YP0224
AKT	Santa cruz	5298
Phospho-AKT	Cell Signaling Technology	4060S
mTOR	Cell Signaling Technology	2983
Phospho-mTOR	Cell Signaling Technology	2974
FOXO3a	Cell Signaling Technology	12829S
Phospho-FOXO3a	Cell Signaling Technology	13129S
MHC	Santa cruz	sc-376157
FBX032	Proteintech	55456-1-AP
TRIM63	Abcam	ab74023
P70(S6K) Antibody	Proteintech	14485-1-AP
phospho-P70S6K	Cell Signaling Technology	9234
Anti-GDF8 / Myostatin	Abcam	ab203076
α-Tubulin	Cell Signaling Technology	3837
Tropomyosin	Santa cruz	sc-58868
Desmin	Santa cruz	sc-23879
GAPDH	Abcam	Ab8245
UT receptor	Abcam	ab156003
4EBP	Proteintech	60246-1-Ig
Phospho-4EBP	Cell Signaling Technology	9455
UbLys48	Millipore	05-1307
UbLys63	Millipore	05-1308
